# Quality control mechanisms that protect nuclear envelope identity and function

**DOI:** 10.1083/jcb.202205123

**Published:** 2022-08-29

**Authors:** Philip J. Mannino, C. Patrick Lusk

**Affiliations:** Department of Cell Biology, Yale School of Medicine, New Haven, CT

## Abstract

The nuclear envelope (NE) is a specialization of the endoplasmic reticulum with distinct biochemistry that defines inner and outer membranes connected at a pore membrane that houses nuclear pore complexes (NPCs). Quality control mechanisms that maintain the physical integrity and biochemical identity of these membranes are critical to ensure that the NE acts as a selective barrier that also contributes to genome stability and metabolism. As the proteome of the NE is highly integrated, it is challenging to turn over by conventional ubiquitin-proteasome and autophagy mechanisms. Further, removal of entire sections of the NE requires elaborate membrane remodeling that is poorly understood. Nonetheless, recent work has made inroads into discovering specializations of cellular degradative machineries tailored to meeting the unique challenges imposed by the NE. In addition, cells have evolved mechanisms to surveil and repair the NE barrier to protect against the deleterious effects of a breach in NE integrity, in the form of either a ruptured NE or a dysfunctional NPC. Here, we synthesize the most recent work exploring NE quality control mechanisms across eukaryotes.

## Teaser

The nuclear envelope surrounds and protects the genome. Mannino and Lusk explore the unique set of quality control pathways that maintain its identity and function.

## Introduction

By housing the genome, the nucleus plays a central role in establishing and maintaining cellular identity and function. Key to this role is the segregation of nuclear and cytosolic contents, which is assured by the integrity of a selectively permeable nuclear envelope (NE) barrier. Indeed, the NE is protective of the genome, as perturbations to its integrity lead to deleterious intermixing of cytoplasm and nucleoplasm and DNA damage (Raab et al., 2016; Earle et al., 2020; Nader et al., 2021). More broadly, a compromised NE barrier has been observed as a normal consequence of cellular and organismal aging (Pathak et al., 2021), which is further exacerbated by disease variants that weaken the biochemical interaction networks that maintain the integrity of the NE (Earle et al., 2020; Kim et al., 2021). Thus, there is a need for quality control mechanisms that act specifically to maintain, and in some cases repair, a defective NE barrier. Recent work is defining such pathways. In so doing, these studies are introducing NE specializations of established endosomal sorting complexes required for transport (ESCRT) membrane sealing mechanisms, in addition to ubiquitin-proteasome and autophagy-mediated degradation that are geared toward meeting the unique topological challenges imposed by the NE.

## NE fundamentals

**Figure 1.**
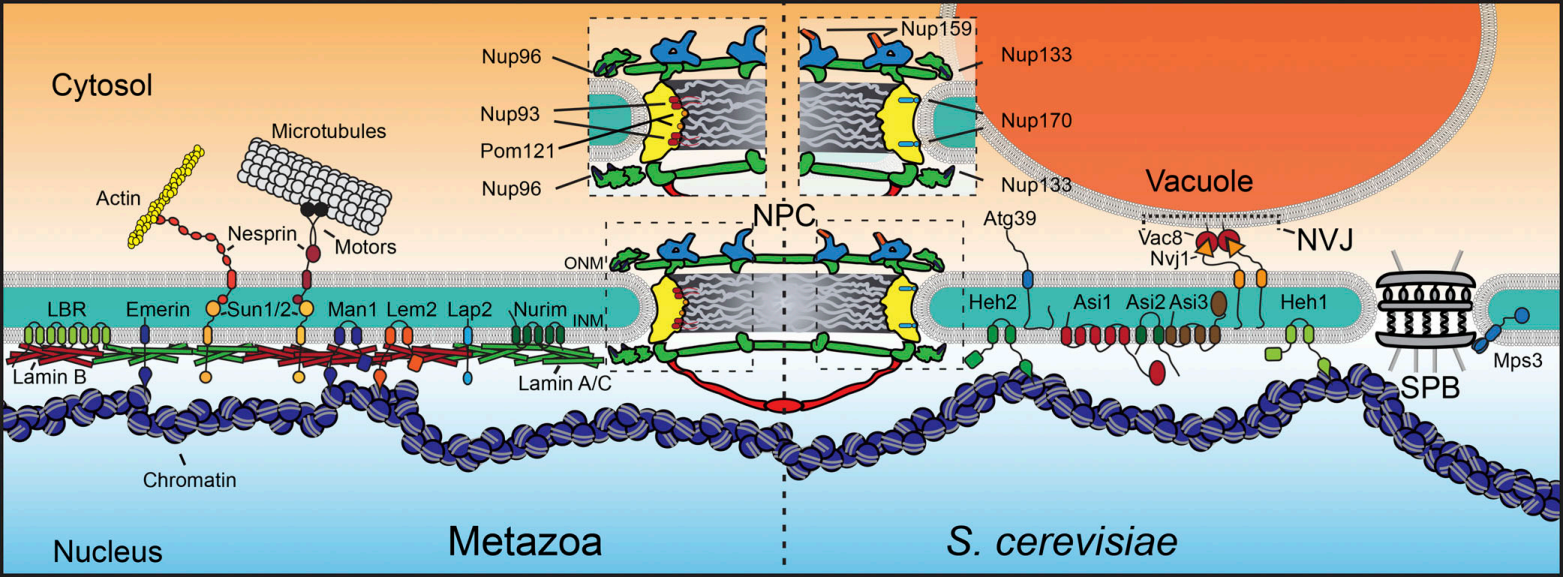
**The NE in metazoans and *S. cerevisiae*.** A schematic of the NE and associated proteins in metazoan (left) and *S. cerevisiae* cells (right). Relative positions of nups mentioned in the text shown within the NPC scaffold, which consists of the inner ring (yellow) and outer ring (green). Cytosolic filaments (blue) and nuclear basket (red) also shown. SPB, spindle pole body.

The NE is made up of a continuous phospholipid bilayer that connects at least three biochemically distinct membranes: the outer nuclear membrane (ONM), the nuclear pore membrane (POM), and the inner nuclear membrane (INM; Fig. 1). The ONM is contiguous with the ER but contains transmembrane proteins that bind to lumenal domains of resident integral INM proteins (Pawar and Kutay 2021) or directly to the lumenal leaflet of the INM itself (Millen et al., 2008; Chandra et al., 2021; Mochida et al., 2022; Fig. 1). The INM is home to several integral and peripherally associated membrane proteins that directly interface with the genome and are often secured to the nuclear lamina (in metazoans), a network of intermediate filaments made up of A- and B-type lamins (Fig. 1). There remains uncertainty surrounding the precise catalogue of integral INM proteins, as any ER membrane protein with small (less than ∼60 kD) extraluminal domains can, in principle, cross the POM. Thus, to define INM residency requires an explicit consideration of the relative enrichment of a given membrane protein at the INM versus the rest of the ER, which should further be supported by functional interactions with the lamina and/or chromatin (Soullam and Worman 1995; Ohba et al., 2004; Ungricht et al., 2015; Smoyer et al., 2016; Cheng et al., 2019; Mudumbi et al., 2020). The INM and ONM are connected by the POM, which delimits ∼100-nm-diameter nuclear pores. The latter are filled with 100-megadalton nuclear pore complexes (NPCs) that establish a diffusion barrier and selective transport channel (Wente and Rout 2010; Schmidt and Gorlich 2016; Wing et al., 2022); alongside the impermeability of the INM and ONM, the selective barrier properties of the NPC establish and maintain nucleocytoplasmic compartmentalization.

## A highly integrated NE protein network is resistant to turnover

The robustness of the NE barrier requires the turnover of its component parts. Intuitively, one can envision that, because of its highly integrated and interdependent nature, the NE proteome may be difficult to turn over. Indeed, the lamins are very long lived, with half-lives that range from 29 h to >100 d depending on the cell type (Mathieson et al., 2018). Further, early studies in *Caenorhabditis elegans* (D’Angelo et al., 2009) and rat brain (Savas et al., 2012; Toyama et al., 2013) revealed that important architectural scaffold components of NPCs (nucleoporins [nups]) are also very long lived, with half lives of months to years. Even in single-cell eukaryotes with division times measured in hours, the turnover of nups is slow (Christiano et al., 2014; Hakhverdyan et al., 2021). Perhaps surprisingly, integral INM proteins have half-lives similar to those of integral ER proteins (Mathieson et al., 2018; Buchwalter et al., 2019), suggesting that mechanisms that control the turnover of ER proteins also function at the INM. Regardless of this wide range of turnover rates among NE components, there is growing evidence that all major components of the NE including individual nups, whole NPCs, the lamins, and integral INM proteins can be removed and degraded by both the ubiquitin-proteasome system (UPS; Box 1) and lysosomes/vacuoles through autophagy (Box 2). These events can be programmed as part of pathways that lead to changes in cell fate but also can be triggered by protein damage and misfolding or in response to cellular stress such as DNA damage or nutrient deprivation. In the following, we examine the current understanding of how the major cellular degradation machineries interface with the NE, with a focus on specializations that enable turnover of this critical subcellular compartment.

Box 1. UPS, ERAD, and INMAD

UPS is one of the major protein degradation mechanisms. Proteins destined for proteasomal degradation are covalently labeled with polyubiquitin chains by a series of enzymatic reactions catalyzed by enzymes categorized as E1 ubiquitin-activating enzymes, E2 ubiquitin-conjugating enzymes, and E3 ubiquitin ligases. Although there are only 2 E1 and 40 E2 enzymes, there are >600 E3 ligases in human cells (Li et al., 2008), which reflects their role in recognizing discrete substrate repertoires. Integral membrane proteins of the ER are also targeted by the UPS but must be removed from the membrane by ERAD ( Mehrtash and Hochstrasser 2019). This is achieved by engaging AAA ATPases such as Cdc48/p97 that provide a pulling force to unfold globular lumenal domains through retrotranslocon channels. INM proteins are targeted by an analogous mechanism (INMAD). Proteins are targeted by either soluble E3 ligases or the INM-resident E3 ligases Asi1 and Asi3, which, together with a substrate adaptor Asi2, comprise the Asi complex. In addition to the Asi complex, Doa10 and likely other retrotranslocons yet to be discovered can help remove integral INM proteins from the lipid bilayer and target them for degradation by the proteasome.

Box 2. Autophagy

Autophagy is required to degrade protein aggregates and parts of or whole organelles such as mitochondria, ER, lipid droplets, peroxisomes, and the nucleus. Autophagy encompasses the recognition, capture, and transport of cargo molecules into the lytic compartment of the cell (lysosomes in metazoans; vacuoles in yeasts and plants). There are three types of autophagy: macroautophagy, microautophagy, and chaperone-mediated autophagy (CMA; Morishita and Mizushima 2019). In macroautophagy, cargo is sequestered into a double-membraned organelle that forms de novo known as an autophagosome (termed “phagophore” before closure; Melia et al., 2020). This process requires a multitude of autophagy (ATG) proteins that control the biogenesis of the autophagosome. Cytoplasmic cargo molecules can be randomly sequestered into the autophagosome (bulk autophagy); alternatively, macroautophagy can recognize and capture specific cargos (selective autophagy). A critical step in autophagosome biogenesis is the covalent labeling of the ubiquitin-like Atg8 family of proteins (LC3 and GABARAP family proteins in mammals; Atg8 in yeast) to the phospholipid phosphatidylethanolamine on the inner and outer membranes of the autophagosome by a series of enzymatic reactions catalyzed by Atg7 (E1 like) and Atg3 (E2 like). Cargo specificity is imparted by cargo adaptor proteins that interact with the cargo and contain binding motifs for Atg8 family members. Cargo adaptor proteins in yeast also bind to the scaffold protein Atg11 (the closest homologue in mammals is FIP200), which then recruits many of the autophagy factors to promote autophagosome assembly around the cargo (Eickhorst et al., 2020). In contrast to macroautophagy, microautophagy and CMA do not involve autophagosomes to deliver cargo to the lysosome/vacuole. In microautophagy, which nonetheless requires many of the core ATG proteins, the lysosome/vacuole invaginates to allow entry of the cargo molecules. By contrast, CMA, which has not been identified in yeast, does not require core ATG proteins. Instead, CMA involves the recognition of a signal peptide on cargo molecules by chaperones. The chaperones unfold the substrate and facilitate its translocation into the lysosome via LAMP2A, which forms a translocon on the surface of lysosomes.

## INM-associated degradation (INMAD) mechanisms protect INM identity

The UPS is key machinery that degrades the majority of soluble and membrane proteins in a cell. ER membrane proteins require additional factors in a dedicated ER-associated degradation (ERAD; Box 1) mechanism that must first evict the protein from the lipid bilayer before it can be targeted by the proteasome. In budding yeast, ERAD is mediated by three integral membrane E3 ligases: Hrd1, Doa10, and the Asi complex, consisting of Asi1, Asi2, and Asi3, two of which (Asi1 and Asi3) have RING domains that define a family of E3 ligases (Forsberg et al., 2001). Hrd1 (Schoebel et al., 2017; Vasic et al., 2020; Wu et al., 2020), and likely Doa10 and the Asi complex (Natarajan et al., 2020; Schmidt et al., 2020) also form channels that enable the retrotranslocation of membrane proteins in concert with the AAA ATPase Cdc48/p97, which is thought to provide the force to unfold the lumenal domain of the substrate to facilitate its retrotranslocation through the channel.

**Figure 2.**
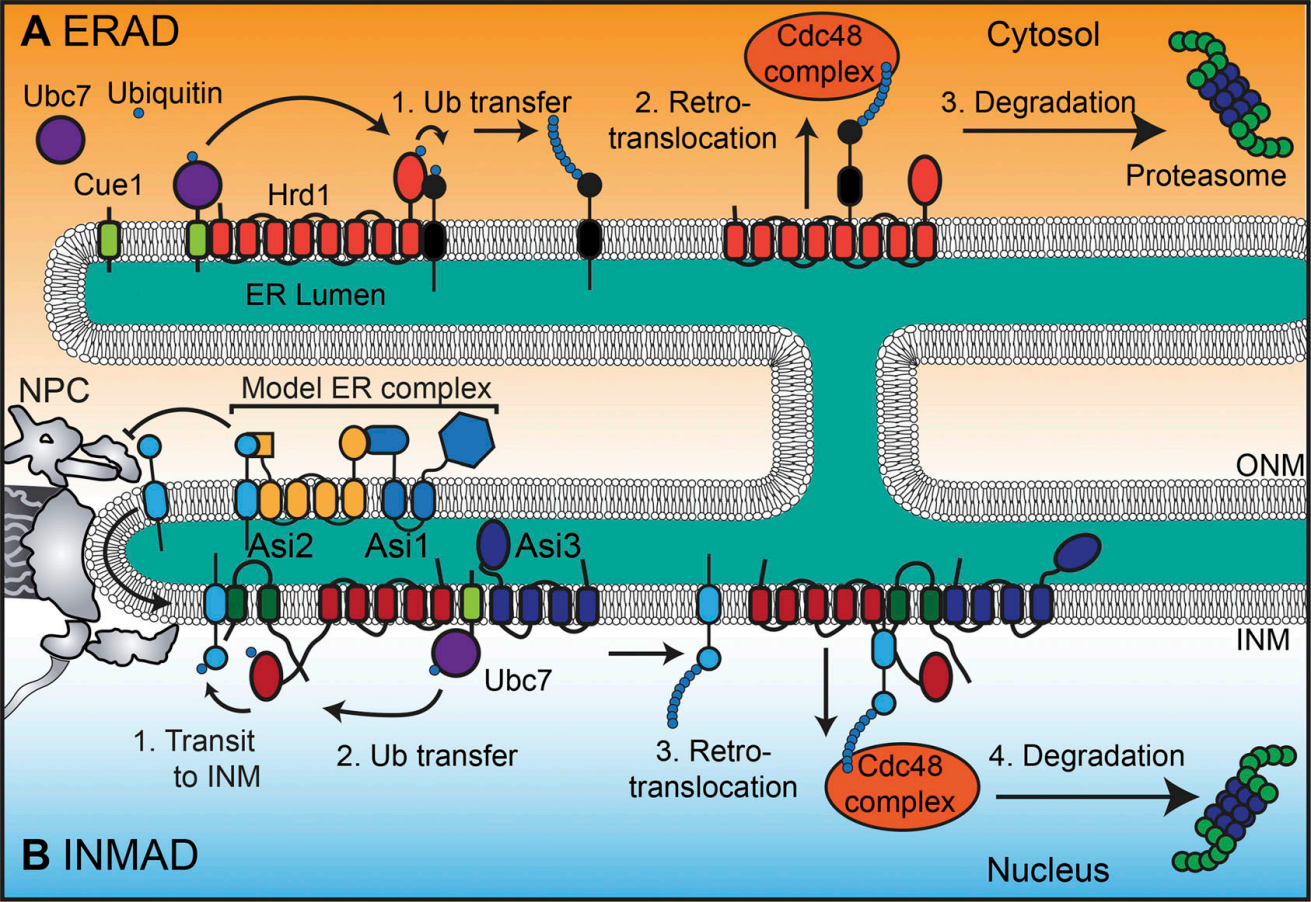
**ERAD and INMAD. (A)** Schematic of ERAD of a hypothetical substrate. (1) Upon binding the substrate, Hrd1 receives multiple ubiquitin moieties (sequentially) from an E2 such as Ubc7 and transfers them to the substrate (black). (2) The substrate is retrotranslocated from the ER membrane into the cytosol in a manner dependent on the Cdc48/p97 complex and a retrotranslocon channel formed by Hrd1. (3) The substrate is recruited to the proteasome, where it is degraded. **(B)** INMAD is required for clearing INM proteins through a mechanism similar to ERAD, but instead of Hrd1, the Asi complex or other E3 ubiquitin ligase/retrotranslocon channels are used. INMAD is also used to degrade orphan subunits (light blue) of ER complexes as depicted: (1) Multiprotein complexes are too large to diffuse past the peripheral channels between the NPC and the POM, but orphan subunits can. (2) Asi2 then binds the orphaned substrate via its transmembrane domain; Asi1 receives a ubiquitin moiety from an E2 such as Ubc7 and transfers it to the substrate. (3) The orphaned substrate is retrotranslocated from the INM into the nucleus in a manner dependent on the Cdc48/p97 complex and a retrotranslocon channel likely formed by the Asi complex. (4) The orphaned substrate is recruited to the proteasome, where it is degraded.

Interestingly, the three budding yeast E3 ligases are uniquely distributed within the NE-ER system: Doa10 is found throughout the ER but can access the INM, Hrd1 is excluded from the INM because it is too big to slip by the NPC scaffold at the POM (Deng and Hochstrasser 2006), and the Asi complex is an INM resident (Boban et al., 2006; Zargari et al., 2007; Fig. 2). Because of its localization, the Asi complex is a major player in ERAD-like INMAD mechanisms that target several substrates, including integral INM proteins (Foresti et al., 2014; Khmelinskii et al., 2014; Smoyer et al., 2019; Table 1). Although a systematic screen leveraging a split-GFP approach has revealed the breadth of the Asi substrate repertoire (Smoyer et al., 2019), there are likely several more INMAD substrates to be discovered, as variations of INMAD requiring distinct machineries are coming to light.

**Table 1.** List of NE proteins demonstrated to be degraded by autophagy or the UPS

NE protein	Species	Degradation mechanism	E3 ligase	Reference
Mps3	*Saccharomyces cerevisiae*	UPS	Anaphase-promoting complex	Koch et al. (2019)
Heh2	*Saccharomyces cerevisiae*	UPS	Asi1	Smoyer et al. (2019)
Per33	*Saccharomyces cerevisiae*	UPS	Asi1	Smoyer et al. (2019)
Asi2	*Saccharomyces cerevisiae*	UPS	Asi1, Doa10	Smoyer et al. (2019); Pantazopoulou et al. (2016)
Asi1	*Saccharomyces cerevisiae*	UPS	Unknown	Pantazopoulou et al. (2016)
Nup85	*Saccharomyces cerevisiae*	UPS	Unknown	Webster et al. (2014)
Heh1	*Saccharomyces cerevisiae*	Nucleophagy	NA	Mochida et al. (2015)
Hmg1	*Saccharomyces cerevisiae*	Nucleophagy	NA	Mochida et al. (2015)
Nur1	*Saccharomyces cerevisiae*	Nucleophagy	NA	Mostofa et al. (2018)
Whole NPCs	*Saccharomyces cerevisiae*	Nucleophagy	NA	Tomioka et al. (2020); Lee et al. (2020)
Nurim	Mouse	UPS	Unknown	Buchwalter et al. (2019)
Nup62	Mouse	UPS	Unknown	Zhu et al. (2016)
Nup153	Mouse	UPS	Unknown	Zhu et al. (2016)
Nup214	Mouse	UPS	Unknown	Zhu et al. (2016)
Nup358	Mouse	UPS	Unknown	Zhu et al. (2016)
Lamin B	Mouse, zebrafish	UPS	Wdr26	Zhen et al. (2020)
β-Dystroglycan	Mouse	UPS	Unknown	Vélez-Aguilera et al. (2018)
Emerin	Mouse, human	UPS (mouse and human), nucleophagy (human)	Unknown	Buchwalter et al. (2019); Muchir et al. (2006); Lenain et al. (2015)
SUN1	*Arabidopsis*, human	UPS (*Arabidopsis*), nucleophagy (human)	Unknown	Huang et al. (2020); Lenain et al. (2015)
SUN2	Human	UPS	Skp1βTRCP1/2	Coyaud et al. (2015); Kim et al. (2015)
LBR	Human	UPS, nucleophagy	Unknown	Tsai et al. (2016); Lenain et al. (2015)
Nup188	Human	UPS	Unknown	Vishnoi et al. (2020)
LAP2α	Human	UPS	RNF123	Khanna et al. (2018)
Lamin B1	Human	UPS, nucleophagy	RNF123	Dou et al. (2015); Khanna et al. (2018); Lenain et al. (2015)

NA, not applicable.

One such INMAD mechanism targets the integral INM protein Mps3, which is degraded by a Cdc48/p97-dependent pathway that does not engage the Asi complex or Doa10. In this case, Mps3 is ubiquitylated by the soluble anaphase-promoting complex (Koch et al., 2019). A similar mechanism may also be used for the plant Mps3 orthologue, SUN1, with an additional level of regulation provided by plant ubiquitin regulatory X domain–containing proteins that stabilize SUN1 perhaps by binding to Cdc48/p97 (Huang et al., 2020). Other examples include SUN2, which is degraded by the proteasome after it is ubiquitylated by the soluble E3 ligase Skp1-Cullin1-F-box-βTRCP1/2 (Skp1^βTRCP1/2^) complex (Coyaud et al., 2015; Kim et al., 2015) and the degradation of a disease-causing form of lamin B receptor (LBR) that also occurs through a Cdc48/p97-dependent mechanism (Tsai et al., 2016). The latter example stands out, however, as a soluble form of the unstable LBR mutant accumulated in the nucleus upon proteasome inhibition, supporting that its degradation does in fact occur within this compartment. Thus, providing clear evidence of INMAD in metazoans and plants requires tools to evaluate the location of proteasome engagement and/or the identification of INM-specific retrotranslocons. Candidates to investigate may be the Derlin family, as theyform retrotranslocons but lack E3 ligase activity (Kandel and Neal 2020).

The existence of INMAD implies that proteasomes must also be found within the nucleus. This has long been understood in yeast (Enenkel et al., 1998; Wilkinson et al., 1999), where there is a dedicated nuclear proteasome import pathway (Wendler and Enenkel 2019), but also holds true for higher eukaryotes (de Almeida et al., 2021). Further, stunning cryo-EM of *Chlamydomonas reinhardtii* nuclei revealed the presence of proteosomes tethered to the INM and the nuclear basket of the NPC (Albert et al., 2017). The proteasome association with NPCs (also indicated by physical interactions with nucleoporins in budding yeast; Niepel et al., 2013) might imply that shunting some misfolded proteins into the nucleus is part of a more general degradation mechanism for ubiquitylated proteins, which has been suggested (Balchin et al., 2016). Indeed, the concept that the nucleus might act as a garbage depot for some proteins is borne out by evidence supporting that mistargeted vacuolar membrane proteins are degraded through a mechanism that depends on their reaching the INM and the Asi complex (Khmelinskii et al., 2014).

That mistargeted membrane proteins can be degraded in an Asi-dependent mechanism further suggests that INMAD executes a form of quality control that ensures that the INM is not polluted by proteins that should not be there. How the Asi complex could perform such a task remains mysterious but speaks to the idea that the Asi complex helps maintain INM identity. This idea extends to compelling evidence where the Asi complex contributes to the degradation of orphan members of ER-resident transmembrane protein complexes such as the oligosaccharyl transferase complex (Fig. 2). Suprastoichiometric components of the oligosaccharyl transferase complex can access the INM, whereas the fully assembled eight-member complex is excluded from transiting the POM (Natarajan et al., 2020; Fig. 2). Thus, the NPC along the POM may act as a filter that allows access of some ER transmembrane proteins, where they are then culled by the Asi complex. This emphasizes the need to fully understand how the NPC controls the translocation of membrane proteins (Ungricht and Kutay 2015), but also how the Asi complex differentiates between bona fide integral INM proteins, those that are mistargeted, and those smaller ER proteins that might benignly sample the compartment (Smoyer et al., 2016). It is possible that Asi2 contributes to these decisions, as it has been demonstrated to recognize the transmembrane domains of a subset of Asi complex substrates (Natarajan et al., 2020), but how Asi2-independent substrates are recognized requires additional study. Further, the decision behind why some substrates such as Pom33 are ubiquitylated, but not necessarily degraded, needs to be addressed, particularly as the latter impacts the distribution of some nups along the NE as well (Smoyer et al., 2019).

## Clearance of nups and NPCs

Although Pom33 might not be degraded by INMAD, there is evidence that essentially all nups, even those deeply embedded in the NPC scaffold, can be removed and replaced, suggesting that they could be targets of UPS. The exchange rate of nups between existing or newly synthesized pools correlates with their position in the NPC structure: central channel and peripheral elements exchange faster than those that construct the core scaffold (Hakhverdyan et al., 2021), with exceptions that may depend on cell type. For example, the use of a recombination-induced tag exchange approach that allows tracking of the exchange of nups in terminally differentiated mouse cells in vitro revealed that individual scaffold nups such as Nup133 and Pom121 exchange rapidly with newly synthesized proteins (Toyama et al., 2019). Others such as Nup93 and Nup96 exchange much more slowly, largely in line with early studies on nup exchange between NPCs in mammalian cells (Daigle et al., 2001). Thus, it remains ill defined why some scaffold nups can turn over while others cannot. Clues to the underlying mechanism could be derived from the yeast system, where whether a given nup can exchange from the NPC depends on the presence of a free pool (Hakhverdyan et al., 2021). Thus, it is possible that when considering whether a given nup is replaced in a specific cell type, the transcriptional regulation of nup genes may be the most important factor, as opposed to the mechanism of nup removal.

Whether a given nup is turned over in the NPC might also depend on whether it loses function due to damage. For example, yeast nups that serve as connectors that tie multiple subcomplexes together exchange faster than those that construct individual subcomplexes (Hakhverdyan et al., 2021; Fig. 1). These data hint that the connectors need to be replaced more frequently, as they may be prone to damage, perhaps because the connectors bear the brunt of the mechanical forces placed on the NPC by tension on the NE; this tension has recently been shown to drive large conformational changes in the NPC scaffold that result in a dilation of the central transport channel (Schuller et al., 2021; Zimmerli et al., 2021; Akey et al., 2022). Consistent with the idea that there are mechanisms to selectively remove damaged nups, the addition of a degron to Nup170, a key component of the inner ring (Fig. 1), led to its rapid removal and degradation and a concomitant increase in its exchange with a degron-less version (Hakhverdyan et al., 2021). Attaching degrons to multiple mammalian nups also leads to their rapid degradation, confirming that the UPS can access the core structure of the NPC across eukaryotes and cell types (Li et al., 2021; Schuller et al., 2021).

## The excision and degradation of whole NPCs

**Figure 3.**
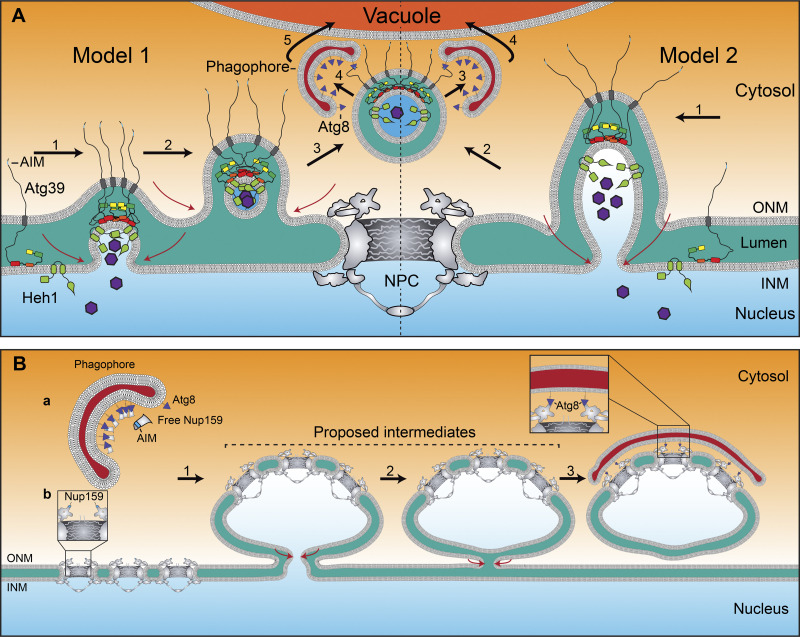
**Proposed models of nucleophagy and NPC-phagy. (A)** Schematic of two proposed models of Atg39-mediated nucleophagy. Model 1: (1) Atg39 binds the INM through its lumenal amphipathic helices (red and orange) and condenses, which contributes to both INM and ONM remodeling to evaginate the INM. (2) An INM fission event (depicted by red arrows) generates an intralumenal vesicle containing nuclear (purple hexagons)/INM cargo (Heh1 as model INM cargo shown). (3) ONM scission releases a double-membrane NE-fragment containing the lumenal vesicle that is (4) recognized by the phagophore membrane by direct engagement between Atg8 and the AIM (blue) of Atg39. (5) The autophagosome fuses with the vacuole, releasing the NE fragment into the lumen of the vacuole. Model 2: (1) Atg39 condensation drives INM and ONM remodeling and the evagination of the INM. (2) Scission of the ONM and INM occur simultaneously, resulting in an NE-derived vesicle that can be (3) captured by a phagophore. (4) The autophagosome fuses with the vacuole, releasing the NE fragment into the lumen of the vacuole. **(B)** (a) A schematic of nucleoporinophagy. A free pool of Nup159 is selectively targeted by the phagophore via the binding of Atg8 to the AIM on Nup159 (blue). (b) A schematic of a proposed model of membrane remodeling during NPC-phagy. (1) NPCs cluster, and the NE herniates toward the cytosol. (2 and 3) In one or two membrane fission steps (red arrows), an NE-derived vesicle with multiple NPCs is released from the nucleus. This structure can be selectively recognized by a phagophore via Atg8 and the AIM on Nup159.

In addition to the degradation of individual nups, there is emerging evidence for the removal and degradation of entire 50–100-megadalton NPCs from the NE. As the proteasome is not designed to degrade massive protein assemblies, cells must turn to autophagy, which is much better suited for the clearance of large protein complexes, aggregates, and pieces of organelles through several general mechanisms (Box 2). Any degradation of whole NPCs would necessarily also involve elaborate membrane remodeling to excise the NPC from the NE without compromising nuclear integrity (Fig. 3).

Evidence for the autophagy of NPCs is found in the budding yeast model under conditions of nitrogen starvation or pharmacological inhibition of the TORC1 kinase (Allegretti et al., 2020; Lee et al., 2020; Tomioka et al., 2020). Indeed, both individual nups (nucleoporinophagy) and whole NPCs (NPC-phagy) can be targeted by autophagy and degraded in lysosomes/vacuoles. The evidence suggests that there is a direct recognition of NPCs by the major autophagy protein Atg8/LC3 through Atg8-interacting motifs (AIMs) in at least Nup159 (Lee et al., 2020; Tomioka et al., 2020), which is ideally positioned on the cytosolic face of the NPC to engage with this cytosolic autophagy machinery (Fig. 3). There remains some debate, however, over whether there is a dedicated NPC-phagy cargo adaptor that is yet to be discovered (Tomioka et al., 2020; Yin and Klionsky 2020), or whether the direct interaction between Atg8 and Nup159 executes only nucleoporinophagy (Tomioka et al., 2020). Regardless, compelling ultrastructural evidence demonstrates that NPCs within segments of the NE are delivered to vacuoles in a mechanism dependent on core autophagy genes (Allegretti et al., 2020; Lee et al., 2020; Tomioka et al., 2020). Future work is needed to fully define the molecular players and underlying NPC selectivity and membrane remodeling mechanisms.

Indeed, it remains unclear whether there are specific NPCs that are targeted by NPC-phagy or whether NPC-phagy is analogous to bulk autophagy mechanisms that are indiscriminate in nature (Dikic and Elazar 2018). Clues that the mechanism is specific for the clearance of damaged/defective NPCs, however, can be found when genetic perturbations to NPC structure by, for example, depleting Nup133 or Nup120, increase NPC-phagy (Lee et al., 2020). Interestingly, the deletion of Nup133 or Nup120 leads to NPC clustering (Doye et al., 1994; Aitchison et al., 1995; Pemberton et al., 1995), conveniently sequestering malfunctioning NPCs into a discrete region of the NE. The latter might facilitate engagement of the defective NPCs by the autophagosome for their ultimate removal (Lee et al., 2020). Conceptually, this is analogous to previously reported mechanisms that cluster misassembled NPCs to prevent their inheritance during asymmetric cell division (Webster et al., 2014) and, more broadly, to spatial quality control mechanisms that function at the NE (Sontag et al., 2017).

Such an NPC clustering model, however, depends on understanding why NPCs missing Nup133 or Nup120 cluster in the first place, which has been suggested to result from a loss of interactions between these (and other) scaffold nups and the POM (Fig. 1; Kim et al., 2014). Another related possibility is that there are molecular sensors of NPC structure and/or function that also help maintain a normal NPC distribution. Heh2/Man1, an integral INM protein of the Lap2-emerin-MAN1(LEM) family, may play such a role, as its biochemical interactions with the NPC are disrupted by deleting several components of the NPC scaffold including Nup133 (Borah et al., 2021). As Heh2 is required for normal NPC distribution (Yewdell et al., 2011; Borah et al., 2021), likely by connecting NPCs to chromatin (Yam et al., 2013), a reasonable hypothesis is that damage to the NPC releases Heh2, leading to NPC clustering and the ultimate targeting of defective NPCs by autophagy—topics for future studies. NPCs are also cleared en masse during budding yeast meiosis. Indeed, meiotic progeny of old mother cells have the same replicative lifespan potential as those that are born from young mothers (Unal et al., 2011). Thus, meiosis executes a rejuvenation program in which many established aging-associated/senescence factors such as protein aggregates, rDNA circles, and NPCs are sequestered away from the spores during anaphase II. These factors are ultimately degraded by “mega-autophagy,” in which the vacuole releases its resident proteases into the cytosol (King et al., 2019). The sequestration of senescence factors away from the newly formed progeny necessarily requires remodeling of the NE and formation of the gametogenesis uninherited nuclear compartment (GUNC; Koch et al., 2020). How the NE is remodeled in this scenario is not clear, but it appears to be coupled with and require de novo synthesis of plasma membrane (King et al., 2019). Moreover, the sequestration of NPCs in the GUNC requires the ESCRT protein Chm7, the LEM domain integral INM proteins Heh1 and Heh2, and the AAA-ATPase Vps4 (Koch et al., 2020), perhaps suggesting that a membrane fission mechanism is involved. Curiously, although NPCs are degraded, components of the nuclear basket are left in the nuclei of the progeny after meiosis II (King et al., 2019). Although the reason for this is unclear, one suggestion is that the nuclear basket may initiate the de novo formation of NPCs in the spores (King et al., 2019), which would be consistent with inside-out mechanisms of interphase NPC biogenesis (Hamed and Antonin 2021).

### Selective degradation of the INM by nucleophagy

In addition to NPCs, there is overwhelming evidence that the INM can be degraded by a form of macroautophagy called nucleophagy in both budding yeast (Mochida et al., 2015) and mammalian cells (Dou et al., 2015; Lenain et al., 2015). Nucleophagy is remarkably selective and is able (for example) to target the INM but not NPCs (Chandra et al., 2021) or remove Lamin B1 networks but not those generated by Lamin A (Dou et al., 2015). These selective cargo recognition events are even more impressive, as they also result in the capture of nuclear components by a cytosolic autophagy apparatus (Box 2). Thus, the cargo cannot be “seen” by the autophagy machinery because it is hidden by the NE itself. However, cells have devised mechanisms that act from both inside and outside of the nucleus to overcome this challenge.

The discovery of an outside-the-nucleus-in nucleophagy mechanism benefited from the identification of Atg39, the only known NE-specific autophagy cargo adaptor (Mochida et al., 2015). Atg39 is a type II transmembrane protein localized specifically at the ONM, with its N-terminus facing the cytosol and its C-terminus extending into perinuclear space/NE lumen (Chandra et al., 2021; Mochida et al., 2022; Fig. 1). Evidence supports that Atg39 accumulates specifically at the ONM by direct binding between amphipathic helices in its lumenal domain with the lumenal leaflet of the INM (Mochida et al., 2022). Thus, specific features of the INM that are preferred by the Atg39 lumenal domain are absent from the broader ER. The underlying mechanism for this specificity remains ill defined, but it has been proposed that because the lumen is wider in the cortical ER than at the NE (West et al., 2011), the lumenal domain may simply be too short to reach across the ER lumen (Mochida et al., 2022). Alternatively, as the ER beyond the NE in budding yeast is predominantly tubular in nature, it is possible that Atg39 prefers membrane sheets akin to sheet-forming ER proteins that also rely on lumenal domains to perform this function (Shibata et al., 2010; Amaya et al., 2021; Wang et al., 2021). It must also be considered that the lumenal leaflet of the INM could have a unique lipid composition that is detected by the lumenal amphipathic helices in the Atg39 lumenal domain. This idea is supported by evidence indicating that the INM might have a unique lipidome that is distinct from that of the ER (Romanauska and Kohler 2018), and there is precedent for amphipathic helices in other proteins recognizing local changes in lipid composition at the NE (Thaller et al., 2021). Future work will be needed to explicitly differentiate between these possibilities.

Interestingly, the amphipathic helices in the Atg39 lumenal domain are required not only for membrane binding, but also to coordinately deform both the INM and ONM, a prerequisite for the ultimate capture of an NE fragment by the autophagosome (Chandra et al., 2021; Mochida et al., 2022). Indeed, Atg39 can drive NE remodeling outside of engagement with the autophagy proteins Atg8 and Atg11 (Box 2) suggesting that on its own, or by recruitment of yet-to-be-defined factors, it can execute early steps of nucleophagy (Chandra et al., 2021). In fact, the overexpression of Atg39 is sufficient to specifically capture integral INM protein cargo, but not other NE structures such as NPCs or spindle pole bodies, into NE blebs (Chandra et al., 2021).

Ultrastructural analysis from two complementary studies supports that both the INM and ONM are remodeled during Atg39-mediated nucleophagy (Chandra et al., 2021; Mochida et al., 2022); however, only one study demonstrated by correlative light and EM that Atg39-rich NE blebs are expansions of the ONM containing ∼100-nm-diameter vesicles derived from the INM (Chandra et al., 2021). Thus, two models of Atg39-dependent nucleophagy must be considered going forward (Fig. 3 A). In one, there is a single membrane scission event that leads to simultaneous fission of the INM and ONM to release an NE fragment (Fig. 3 A, Model 2). In the other, there are two distinct membrane fission steps: one that executes INM fission to generate an intralumenal vesicle, and a second that drives ONM fission to release the NE fragment with INM vesicles enclosed (Fig. 3 A, Model 1). Although each would result in the clearance of INM, the latter might be more effective at protecting the integrity of the NE during these extensive NE remodeling events.

The lumenal vesicle model is also attractive because it provides additional support for the existence of NE egress or NE budding pathways that were first described as a mechanism to export “mega-ribonucleoproteins” from the nuclei of neurons in developing *Drosophila* (Speese et al., 2012; Jokhi et al., 2013). Indeed, analogous NE lumenal vesicles have been observed in electron micrographs of multiple model systems, but a molecular signature is often lacking, so it is difficult to draw direct parallels between these phenomena (Thaller and Lusk 2018). As recent evidence supports that intralumenal vesicles are prevalent in the context of cell stress (Panagaki et al., 2021), it will be important to assess whether they are a product of nucleophagy as well. Such an idea is consistent with the observation that *ATG39* is expressed only under conditions of nitrogen starvation and the triggering of stress response pathways (Vevea et al., 2015; Mizuno and Irie 2021). A nuclear egress model might also be tailored to efficiently sort INM proteins into a vesicle, as this process resembles how membrane protein sorting occurs during endocytosis and the formation of intralumenal vesicles during multivesicular body biogenesis (Larios et al., 2020). Indeed, endocytic mechanisms that incorporate liquid–liquid phase separation/condensation (Zhang et al., 2019) or membrane curvature–induced sorting (Day and Stachowiak 2020) might provide useful conceptual models for exploring the underlying mechanism of cargo accumulation at the INM during nucleophagy, which remains a key outstanding question.

Although it is possible to consider how a lumenal connection between the ONM and the INM could drive membrane bending at the INM, which might itself contribute to cargo sorting, how soluble nuclear components could be selectively incorporated into the INM evaginations remains a mystery (Fig. 3 A). We suggest that it is most likely that integral INM proteins are key intermediaries between the evaginated INM and Atg39 and thus directly contribute to the selection of intranuclear components for autophagic degradation. A likely candidate is Heh1 (aka Src1), the budding yeast orthologue of LEM2. Heh1 is particularly attractive because it recruits ESCRTs to the NE (Webster et al., 2016; Gu et al., 2017; Thaller et al., 2019), which are obvious candidates that might execute the INM fission mechanism. The latter idea would reinforce the conceptual parallel to multivesicular body biogenesis, but the involvement of ESCRTs in nucleophagy is speculation.

The involvement of proteins in the endocytic system for INM protein degradation may go beyond ESCRTs. For example, ER stress causes the translocation of Emerin from the INM to the ER, where it is then sorted into coat protein complex II–coated vesicles (Buchwalter et al., 2019). These vesicles are transported to the Golgi (Buchwalter et al., 2019) and secreted to the plasma membrane before being endocytosed and delivered to lysosomes for degradation. Interestingly, this unorthodox (for INM proteins) degradative mechanism requires interactions between the Emerin LEM domain and yet-to-be-defined factors (Buchwalter et al., 2019). It is possible that a similar mechanism has been observed in diseased neurons with defects in autophagy where Lamin B1 is excreted from cells (Baron et al., 2017), but the underlying mechanisms remain to be fully defined.

There is also evidence that Lamin B1 is degraded by an inside-the-nucleus-out autophagy mechanism that is triggered by oncogene expression. Specifically, the overexpression of HRASV12, a potent cancer driver, induces a cellular senescence pathway that requires the autophagic degradation of Lamin B1 (Dou et al., 2015). Unlike Atg39-dependent nucleophagy, the degradation of Lamin B1 is thought to begin with the direct recognition of Lamin B1 by LC3, the Atg8 orthologue, in the nucleus. Although there is compelling evidence that Lamin B1 can be selectively removed from the NE and delivered to lysosomes for degradation, how the Lamin B1-LC3 complex transits the NE remains obscure. In particular, the predicted evagination of the INM with the Lamin B1-LC3 complex would result in its enclosure in a double membrane, which would effectively hide it from recognition by the phagophore. One possibility is that the INM with LC3-Lamin B1 could fuse with the ONM, essentially leading to an inversion of the INM, which would now face the cytosol. Such a mechanism has been proposed, as it again closely resembles NE egress pathways (Rose and Schlieker 2012). An alternative model would invoke an ONM cargo adaptor analogous to Atg39, which awaits discovery.

## Piecemeal microautophagy of the nucleus

Another selective nuclear autophagy pathway, piecemeal microautophagy of the nucleus (PMN), has, so far, only been described in budding yeast. In this mechanism, a nuclear-vacuole contact site (the nucleus-vacuole junction [NVJ]; Fig. 1) evaginates and pinches off an NE fragment with nuclear contents directly into the vacuole. This mechanism requires an ONM protein, Nvj1, that (like Atg39) acts as an adaptor that links the vacuole (by binding to Vac8) to the INM through its lumenal domain, thought to directly engage the lumenal leaflet of the INM through a likely amphipathic helix (Pan et al., 2000; Millen et al., 2008). Although this mechanism is considered distinct from the macroautophagic nucleophagy pathway, it nonetheless has many similarities: (1) it is triggered by similar inputs such as nitrogen starvation and the inhibition of TORC1 kinase (Roberts et al., 2002); (2) *ATG39 *and other core autophagy genes are required for its execution (Krick et al., 2008; Otto and Thumm 2021); and (3) many proteins that are degraded by PMN are also targeted by nucleophagy (Mochida et al., 2015; Mostofa et al., 2018). Thus, it may be more accurate to portray PMN and nucleophagy as two sides of the same coin, as they act redundantly to degrade similar sets of proteins, lipids, and nucleic acids. Consistent with this idea, the inhibition of both PMN and nucleophagy results in a synthetic loss of fitness in the context of nitrogen starvation (Tasnin et al., 2021). Although PMN has not yet been discovered in metazoans, there are examples of lysosomes in contact with the nucleus (Park et al., 2009; Mutvei et al., 2020). Further, lysosome–ER contact sites have been described, some of which host lipid transfer proteins whose yeast orthologues are found at NVJs (Melia and Reinisch 2022). Thus, in our opinion, it is only a matter of time before PMN-like mechanisms are discovered in higher eukaryotes.

## ESCRT-dependent surveillance of the NE barrier

Ultimately, defining nuclear autophagy mechanisms will require an understanding of the membrane remodeling machineries that act at the NE. Of particular interest will be those capable of performing membrane fission reactions, with ESCRT proteins being prime candidates. Indeed, early studies that defined a quality control mechanism that acts to prevent defective NPC biogenesis demonstrated a critical role for the recruitment of ESCRTs to the NE (Webster et al., 2014). Further, genetic evidence supports that they function both during NPC-phagy (Lee et al., 2020) and in NPC removal mechanisms that do not depend on autophagy factors (Toyama et al., 2019). Indeed, the ESCRTs play a general role beyond turnover mechanisms in protecting the nucleus from losses in nucleocytoplasmic compartmentalization (Gatta and Carlton 2019; Lusk and Ader 2020).

The ESCRT III proteins form polymers that remodel membranes and—with the help of the AAA ATPase, Vps4—drive membrane fission (McCullough et al., 2018; Stoten and Carlton 2018; Vietri et al., 2020a). In the context of the NE, the ESCRT proteins contribute to sealing NE holes that arise due to defective NPC biogenesis (Webster et al. 2014; Webster et al. 2016; Thaller et al. 2019; Thaller et al. 2021) or NE ruptures (Denais et al., 2016; Raab et al., 2016; Gu et al., 2017) and during the normal course of NE reassembly at the end of an open mitosis (Olmos et al., 2015; Vietri et al., 2015; Gu et al., 2017). Interestingly, a dedicated ESCRT, Chm7/CHMP7, is cytosolic, and although it can cross the NPC diffusion barrier, it is actively exported from the nucleus by the recognition of its nuclear export signals by Xpo1/Crm1 (Thaller et al., 2019; Vietri et al., 2020b). The export of Chm7/CHMP7 assures that it does not bind to the integral INM protein Heh1/LEM2—which activates Chm7 polymerization (Thaller et al., 2019; von Appen et al., 2020)—at the wrong time (Vietri et al., 2015; Thaller et al., 2019; Gatta et al., 2021). Indeed, the spatial segregation of Heh1/LEM2 and Chm7/CHMP7 on either side of the NE barrier sets up a surveillance system that is found in a poised state (Thaller et al., 2019; Vietri et al., 2020b). Perturbations to the NE barrier, either to NE membranes by (for example) mechanical disruption or in the course of an aberrant NPC assembly event that forms an NE hole without an effective diffusion barrier, leads to the meeting of Chm7/CHMP7 and Heh1/LEM2 and the activation of membrane remodeling and NE sealing. The latter likely requires the recruitment of additional ESCRT III proteins and Vps4. Recent work also implicates local accumulation of phosphatidic acid as an early signal that helps recruit Chm7 to sites of NE discontinuity (Thaller et al., 2021).

## INM pruning

Curiously, although Chm7/CHMP7 is actively exported from the nucleus in budding yeast and human cells, in *C. elegans*, it is constitutively found at the INM, bound to at least two LEM-domain integral INM proteins (Shankar et al., 2022). Thus, binding to LEM proteins might not activate Chm7/CHMP7 polymerization as it does in yeast and mammals. In fact, in these model systems, allowing Chm7/CHMP7 to bind Heh1/LEM2 in the nucleus drives the formation of a network of fenestrated INM cisterna that are likely deleterious to cell viability (Thaller et al., 2019; Vietri et al., 2020b). In contrast, similar but morphologically distinct tubular INM extensions are also described in *C. elegans*, but in this case, they are a product of CHMP7 loss, not gain, of function (Shankar et al., 2022). The implication is that CHMP7 is required to prune these INM tubules, which might be a byproduct of NE reformation mechanisms at the end mitosis (Penfield et al., 2020). Indeed, similar INM extensions have been observed in rapidly dividing *Drosophila* embryos in the context of incorporating NPCs within annulate lamellae into the NE (Hampoelz et al., 2016).

It is also worth noting that intranuclear extensions of INM have been described in some mammalian cell types as part of a “nucleoplasmic reticulum” (Drozdz and Vaux 2017). Whether these intranuclear membranes arise due to ESCRT loss of function remains to be understood, but there are clues that this might be the case (Arii et al., 2018). Thus, it is possible that the ESCRTs also execute a kind of INM quality control that prunes aberrant INM tubules that arise during certain NE remodeling events. This idea would necessitate that ESCRTs remodel positive-curvature membranes as opposed to the more typical negative-curvature membranes. As there is evidence to support that ESCRTs can form polymers that bind to positive curvature and might execute such membrane scission reactions (Allison et al., 2013; McCullough et al., 2015; Mast et al., 2018; Bertin et al., 2020), this is a reasonable possibility.

## Outlook

The discovery of NE-specific quality control pathways has introduced new and unexpected mechanisms that require both dedicated NE machineries but also the coopting of proteins initially understood in the endocytic system. With a few exceptions, these pathways have been first characterized in the budding yeast system. Beyond facile genetics, we suspect that the major feature of budding yeast enabling these discoveries is that it undergoes a closed mitosis during which the NE does not breakdown. Thus, yeast have necessarily evolved pathways that can clear NE and nuclear components without compromising nuclear integrity. It follows then that the field may need to look to postmitotic model systems to discover the still-elusive metazoan NE-autophagy cargo adaptor and potential INM E3 ligases. Consistent with this idea, work in induced pluripotent stem cells derived from spinal neurons of amyotrophic lateral sclerosis patients has uncovered that aberrant nuclear entry of CHMP7 is an initiating event that triggers an NPC injury cascade thought to contribute to disease pathogenesis (Coyne et al., 2021). Further, the turnover of disease-causing lamin mutants may keep them at bay and inhibit disease progression (Cao et al., 2011; Pellegrini et al., 2015; Aveleira et al., 2020). Thus, these future discoveries will not only open up new avenues to explore fundamental mechanisms, but they will almost certainly inform disease mechanisms as well. As modern genomics is uncovering more disease variants of integral NE protein and nucleoporin genes, the mechanisms that cells use to clear the resulting aberrant proteins will likely be central to maintaining cell function but also will inform future therapeutic strategies.
